# An Electromagnetic/Capacitive Composite Sensor for Testing of Thermal Barrier Coatings

**DOI:** 10.3390/s18051630

**Published:** 2018-05-19

**Authors:** Yuan Ren, Mengchun Pan, Dixiang Chen, Wugang Tian

**Affiliations:** College of Intelligence Science and Engineering, National University of Defense Technology, Changsha 410073, China; pmc_nudt@vip.163.com (M.P.); chendixiang@163.com (D.C.)

**Keywords:** thermal barrier coatings, eddy current, planar capacitor, composite sensor

## Abstract

Thermal barrier coatings (TBCs) can significantly reduce the operating temperature of the aeroengine turbine blade substrate, and their testing technology is very urgently demanded. Due to their complex multi-layer structure, it is hard to evaluate TBCs with a single function sensor. In this paper, an electromagnetic/capacitive composite sensor is proposed for the testing of thermal barrier coatings. The dielectric material is tested with planar capacitor, and the metallic material is tested with electromagnetic coils. Then, the comprehensive test and evaluation of thermal barrier coating system can be realized. The sensor is optimized by means of theoretical and simulation analysis, and the interaction between the planar capacitor and the electromagnetic coil is studied. The experimental system is built based on an impedance analyser and multiplex unit to evaluate the performance of the composite sensor. The transimpedances and capacitances are measured under different coating parameters, such as thickness and permittivity of top coating as well as bond layer conductivity. The experimental results agree with the simulation analysis, and the feasibility of the sensor is proved.

## 1. Introduction

In order to elevate the thermal efficiency of the engine, it is necessary to improve the temperature of the combustion chamber. The thermal barrier coating (TBC) technology is a surface protection technology developed to meet the high temperature operating state of the engine hot end parts [1]. The conventional TBC structure includes a ceramic layer with low thermal conductivity and a cushioning metal bonding layer. It can not only be used in the aviation field, but also in ship gas turbines, as well as energy and other fields.

With the extensive application of TBCs, its life span is concerned as well. Once the peeling and break occurs in the TBCs, the hot end parts of the engine will expose directly to high temperature environments, and the results are very serious. Therefore, it is very important to evaluate the performance of the TBCs during service.

Several non-destructive evaluation (NDE) techniques for the assessment of TBCs have been developed, mainly including infrared thermograph, acoustic emission, ultrasonic, impedance spectroscopy, microwave and eddy current testing. An infrared thermograph is widely used to assess TBCs [2,3,4,5,6], which can be employed to test not only the thickness of the ceramic top coating but also the porosity content and thermal conductivity of the TBCs. Palumbo and Tamborrino [7] used an empirical thermographic method to evaluate the thickness of the thermal coating and to discriminate between an unevenness of the thickness and a defect zone. Park [8], Renusch [9] and Yang [10] used the acoustic emission technique to test the thermal fatigue damage of the TBCs during the thermal cycle. The results show that the acoustic emission technique can test the thermal growth oxide and micro cracks of the TBCs. The initiation and propagation of the crack in the TBCs can be detected with AC impedance spectroscopy [11,12], which is sensitive to defects such as cracks within yttria-stabilized zirconia coatings and growth of thermally grown oxide (TGO) film between the top coating and the bond coating. Ultrasonic technology was used to investigate the TGO layer, the voids, delamination and porosity in TBCs [13,14,15,16]. Microwave was used to investigate the feasibility of TBC durability monitoring and non-destructive evaluation of surface crack on the substrate beneath the TBCs [17,18]. Khan [19] and Sabbagh [20] used the eddy current testing method to evaluate the life of TBCs and measure its thickness. Fahr [21] and Roge [22] used an eddy current combined with ultrasound methods to evaluate the performance of TBCs.

It can be seen from the above introduction that almost all of the existing methods can only test and evaluate a part of the TBC system. In order to achieve comprehensive evaluation of TBCs, JENTEK Corporation (Waltham, MA, USA) proposed a method combining Meandering Winding Magnetometer (MWM) sensor with Interdigitated Electrode Dielectrometer (IDED) sensor [23]. However, due to the two sensors not having been integrated, the test effect needs to be improved. This paper proposes a composite sensor to test the TBCs, which is based on the principle of eddy current and planar capacitor. In the composite sensor, eddy current testing is used to detect the conductive layer in the TBC system, and planar capacitive testing is used to detect the nonconductive layer in the TBC system. Compared with traditional MWM and IDED sensors, this composite sensor can be utilized to obtain abundant transimpedance and capacitance information from exactly the same test region because of its integrated structure. Information fusion under the two operating modes and different frequencies can be implemented to evaluate the thermal barrier coating accurately and comprehensively.

## 2. Principle of Electromagnetic/Capacitive Composite Sensor

Typically, the TBC is composed of dielectric top coating (TC) and metallic bond coating (BC). The TC is a thin ceramic layer with low thermal conductivity. It is located on the metallic substrate using either plasma spraying or electron beam physical vapor deposition processes. The BC is fabricated to minimize the thermal expansion mismatch between the metallic substrate and the ceramic coating, which is a layer of modified aluminide alloy such as MCrAlY (where M is Co, Fe, Ni, or a mixed combination) [24,25]. It serves to improve the bonding of the ceramic to metallic substrate and to protect the substrate from oxidation. The thickness of the BC is typically 75–150 µm, and the TC is in the range of 80–500 µm. It can be understood that the TBC is a complex coating system that contains both a nonconductive ceramic layer and conductive metal layer, which is hardly to be evaluated with a single method. The combination of electromagnetic and planar capacitive testing can provide a feasible approach for comprehensive assessment of the TBCs.

Electromagnetic testing is a non-destructive testing method for conductive materials, which is based on the Faraday’s law of electromagnetic induction. The damnification in material under test (MUT) can be judged with the impedance change of the induction coil. Within the service cycle of the TBCs, the thickness of the ceramic layer or metal bonding layer may change, and cracks may appear in the metal bonding layer or the superalloy substrate. These phenomena will lead to impedance change of the electromagnetic coils.

Planar capacitive testing is based on the edge effect of electric field, which is used to test the property of dielectric material beneath the planar capacitor. Within the service cycle of the TBCs, the degradation or shedding of the ceramic layer will result in permittivity change, which can be tested with a planar capacitor.

**Figure 1:**
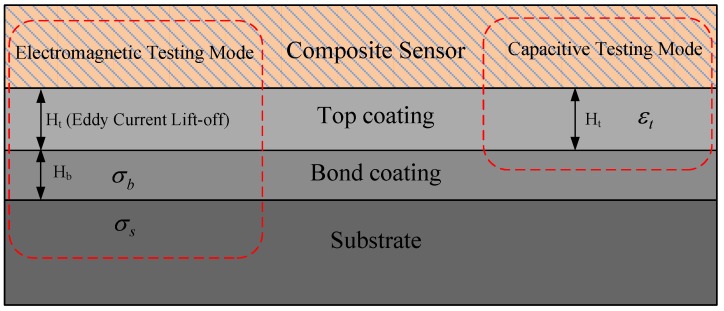
Schematic diagram of thermal barrier coating (TBC) system testing with composite sensor.

Based on the above principles, this paper presents a composite sensor to test either conductive or dielectric materials in the TBCs, which has two different operating modes: electromagnetic testing mode and capacitive testing mode. A representative scenario showing the unknown properties to be estimated by such a hybrid method is shown in Figure 1. In the electromagnetic testing mode, the output signals of the sensor are affected by the thickness of the top coating (*H_t_*) and the bond coating (*H_b_*) as well as the conductivities of the bond coating and the substrate (*σ_b_* and *σ_s_*, respectively). In the capacitive testing mode, the output signals of the sensor are determined by the thickness and the permittivity of the top coating (*H_t_* and *ε_t_*, respectively). Information fusion under the two operating modes can be implemented to evaluate the thermal barrier coating accurately and comprehensively.

## 3. Design of the Composite Sensor

**Figure 2:**
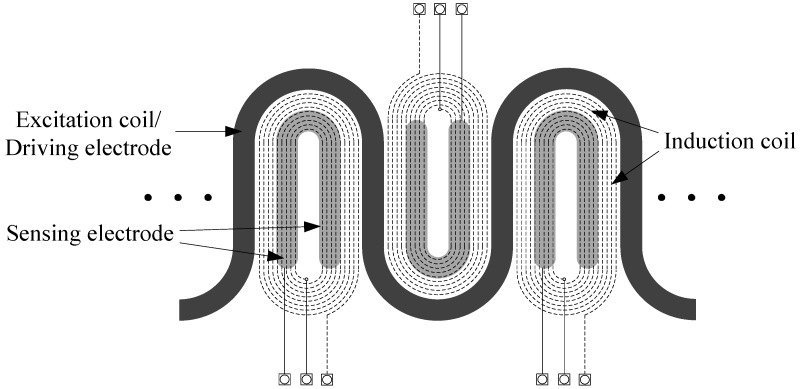
Schematic diagram of the planar composite sensor.

As shown in Figure 2, in the composite sensor, a spatially periodic winding serves as both the excitation coil of the electromagnetic unit and the driving electrode of the planar capacitor. The excitation coil is a meandering structure, which can produce a more uniform electromagnetic field in the material under test (MUT). The multi-turn induction coils locate close to the excitation coil, which is designed to detect the change of eddy current field. The sensing electrode of the planar capacitor is designed as a U-shaped structure, which can increase the equivalent capacitance and testing sensitivity of the planar capacitor.

### 3.1. Finite Element Modeling and Analysis

**Figure 3:**
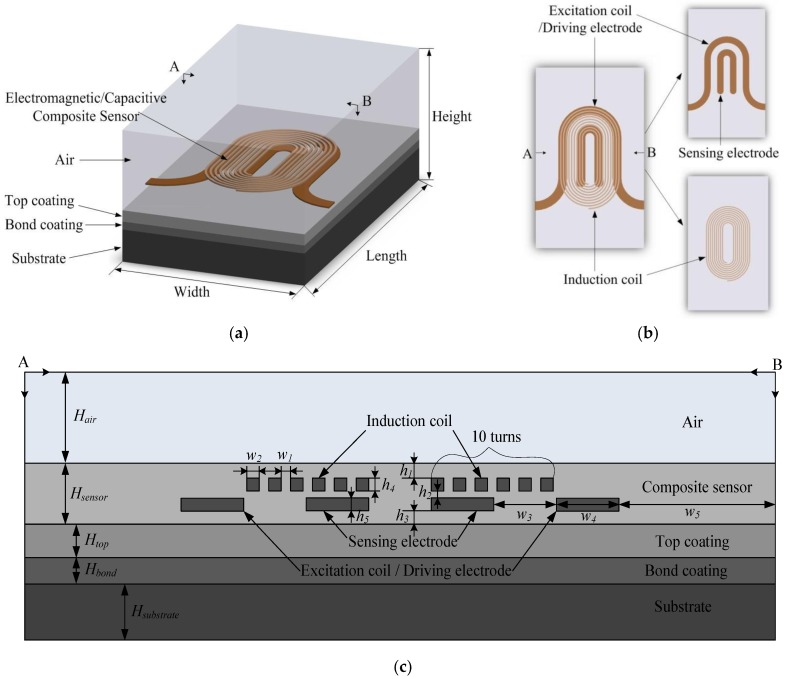
Simulation geometry model. (**a**) overall structure; (**b**) vertical view of overall structure; (**c**) cross-section of composite sensor for a half wavelength, where *H_air_* = 5 mm; *H_sensor_* = 0.116 mm; *H_top_* = 0.3 mm; *H_bond_* = 0.1 mm; *H_substrate_* = 2 mm; *h_1_* = *h_3_* = 0.0275 mm; *h_2_* = 0.025 mm; *h_4_* = *h_5_* = 0.018 mm; *w_1_* = *w_2_* = 0.1 mm; *w_3_* = *w_4_*= 1 mm; *w_5_* = 2.5 mm.

In order to evaluate the performance of the composite sensor, a three-dimensional simulation model has been established using COMSOL Multiphysics 5.2a software (COMSOL Inc., Stockholm, Sweden). In the electromagnetic testing mode, the physical field was selected as magnetic field under AC/DC module and solved with frequency domain solver. In the capacitive testing mode, the physical field was selected as electrostatic field under AC/DC module and solved with stationary solver. The geometric dimension of the whole simulation area is 20 mm (length) × 12 mm (width) × 7.516 mm (height), in which the material under test is the TBC, as shown in Figure 3. The area over the composite sensor is set to be air with the thickness, conductivity, relative permeability and relative permittivity of 5 mm, 0 MS/m, 1 and 1, respectively. The parameters of each layer in the TBC system are listed in Table 1.

The composite sensor consists of a two-layer structure, as shown in Figure 3b. The vertical distance is 25 μm between the induction coil plane and the excitation coil plane. The material of the coil and electrode is set to be copper, whose electrical conductivity, relative permeability and relative permittivity is 59.98 MS/m, 1 and 1, respectively. Apart from the coil and electrode, the material of other part of the sensor is to set to be polyimide, whose electrical conductivity, relative permeability and relative permittivity is 0.004 S/m, 1 and 4 respectively. In the simulated model, the liftoff between the top coating and the composite sensor was not considered. Since the thickness of the wire and the electrode in the sensor is very small, it is set to be a surface without thickness in the physical field of electromagnetic, which can reduce the difficulty of grid dividing and shorten the computation time.

### 3.2. Structure Optimization of the Composite Sensor

**Figure 4:**
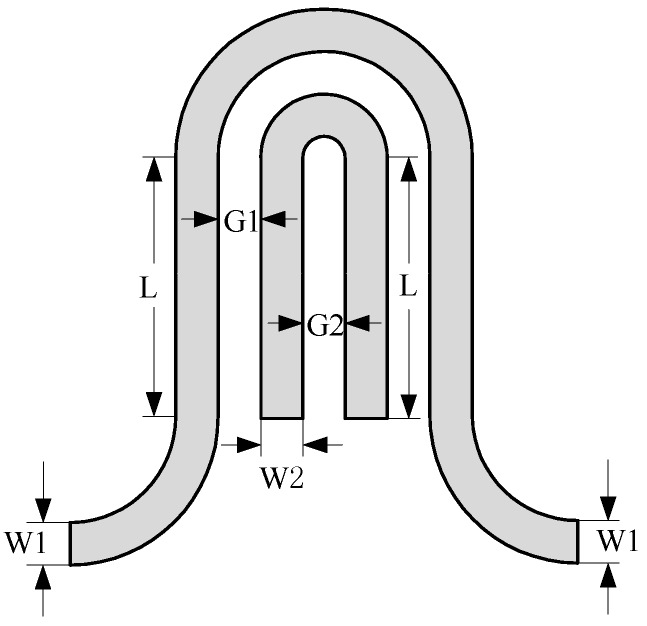
Structure of planar capacitor.

In order to improve the testing performance, it is necessary to optimize the composite sensor. In the capacitive testing mode, the width and spacing of electrodes have great influence on the sensitivity of the sensor. One of the units in the arrayed sensor is shown in Figure 4, in which *W*1 is the width of the driving electrode, *W*2 the width of the sensing electrode, *G*1 is the spacing between the driving electrode and the sensing electrode, *G*2 is the distance between the two ends of the sensing electrode, and *L* is the length of the linear segment of the electrode. In order to maximize the sensitivity, the parameters of each part of the planar capacitor need to be optimized, and the initial parameters are selected as: *W*1 = *W*2 = *G*1 = *G*2 = 1 mm, *L* = 5 mm.

**Figure 5:**
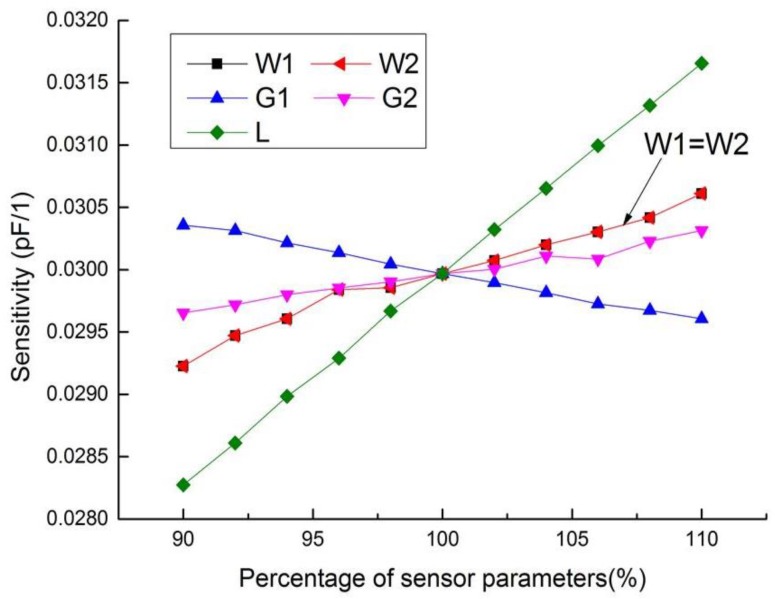
Influence of geometrical parameters on the sensitivity of capacitive unit.

The sensitivity of the planar capacitor is defined as:

(1) $$S_{sensitivity\_C}=\frac{\Delta C}{\Delta \varepsilon },$$

where *ΔC* represents the change of capacitance, and *Δε* represents the change of relative permittivity. It can be seen from Figure 5 that the capacitive testing sensitivity will decrease with the spacing between the driving electrode and the sensing electrode (*G*1) while increase with other parameters, especially the width of the driving electrode and the sensing electrode. However, small electrode spacing of planar capacitor will result in low penetration depth of electric field [26], and the increase of the other parameters will enlarge the size of the testing unit, leading to decrease of spatial resolution. Thus, the trade-off should be made among the testing sensitivity, penetration depth and spatial resolution.

### 3.3. Performance Analysis of the Composite Sensor

When testing the thermal barrier coated blades with an electromagnetic/capacitive composite sensor, either the sensor output or its change caused by coating defect is very small and easy to be affected by other factors. Therefore, it is necessary to analyze the interaction between the induction coil and the sensing electrode of the planar capacitor.

#### 3.3.1. Influence of the Sensing Electrode on the Electromagnetic Testing

**Figure 6:**
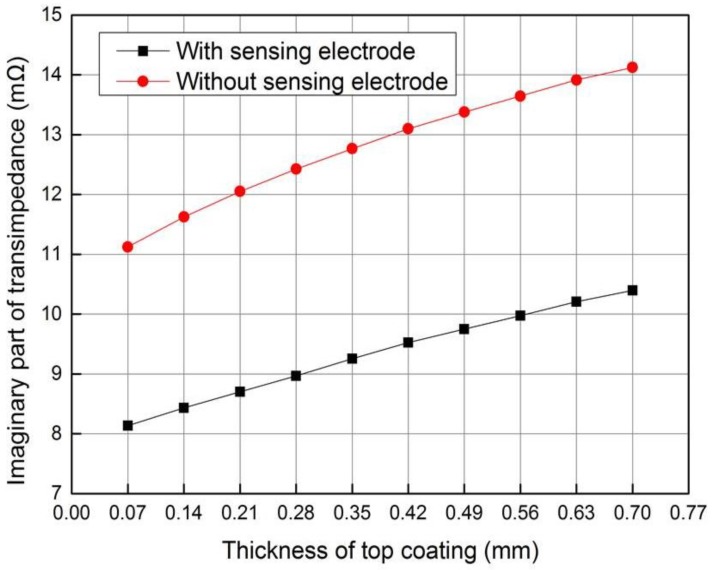
Variation of transimpedance imaginary part with top coating thickness.

In the electromagnetic testing mode, the transimpedance of electromagnetic coil is used to represent the property of sensor, which is defined to be the ratio of induced voltage and excitation current:

(2) $$Z_{transfer}=\frac{V_{S}}{I_{D}},$$

where *I_D_* is the excitation current flowing in the excitation coil, and *V_S_* is the induced voltage of the induction coil. When 20 mA current with 100 kHz frequency is applied to the excitation coil, and the thickness of the ceramic layer is changing from 0.07 mm to 0.7 mm, the transimpedance (Figure 6) can be calculated with Equation (2).

As the thickness of the ceramic layer increases, the eddy current induced on the metal bond coating and substrate will become weaker. Thus, the intensity of secondary magnetic field induced by eddy current will decrease, whose direction is opposite to the main magnetic field generated by the excitation coil. Because the excitation current and main magnetic field intensity remain constant, the total magnetic field strength will increase, and the induced voltage *V_S_* as well as the module of the transimpedance will increase accordingly. Since the imaginary part of the transimpedance is the main concern in electromagnetic induction [27], it is mainly studied in the following simulation and experiment.

It can be seen from Figure 6 that the imaginary part of the transimpedance is approximately in linear relationship with the thickness of the ceramic layer when the sensor contains sensing electrode. However, the relationship is nonlinear when the sensor is working without a sensing electrode. As shown by the slopes of the curves in the Figure 6, the measurement sensitivity of coating thickness is 3.588 Ω/m when the sensor with sensing electrode, smaller than 4.763 Ω/m when the sensor without sensing electrode. The reason is that the eddy current induced on the sensing electrode will influence the eddy current induced on the bond coating and substrate. However, the improvement of the thickness testing sensitivity is achieved in the capacitive testing.

**Figure 7:**
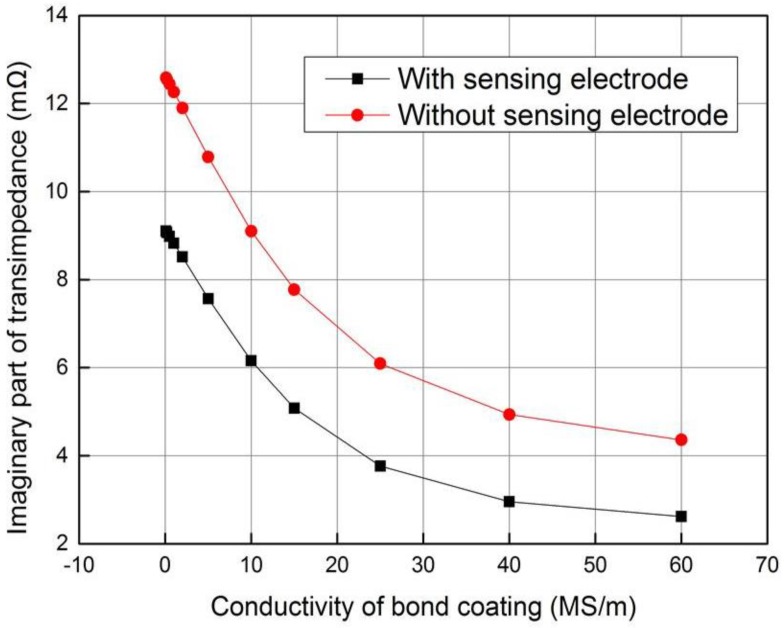
Variation of transimpedance imaginary part with conductivity of bond layer.

The electromagnetic simulations were carried out under eleven different conductivity values of the bond coating: 0.1, 0.2, 0.5, 1, 2, 5, 10, 15, 25, 40, and 60 MS/m. The operating frequency is 100 kHz, as same as in the earlier case. It can be seen from Figure 7 that the imaginary part of transimpedance will fall with the conductivity of bond layer. When the conductivity of the bond coating increases, the eddy current induced on the metal bond coating and substrate will become stronger. Thus, the intensity of secondary magnetic field induced by eddy current will increase, which will reduce the total magnetic field strength, finally leading to the decrease of induced voltage *V_S_* and the imaginary part of transimpedance. When the sensing electrode exists in the sensor, the eddy current will be induced on the sensing electrode and the eddy current on the metal bond coating is smaller. In general, the sensing electrode will influence the sensitivity of the composite sensor more or less under the electromagnetic testing mode.

#### 3.3.2. Influence of the Induction Coil on the Capacitive Testing

In the capacitive testing mode, when a given voltage *U* is applied between the driving electrode and the sensing electrode of planar capacitor, a certain amount of charge *Q* will produce on the two electrodes. The capacitance can be calculated using the equation *C* = *Q*/*U*. When the physical properties of the dielectric materials inside the planar capacitor change, the electric field distribution around the planar capacitor will also change, thus affecting the charge produced on the electrodes and the capacitance of the planar capacitor.

**Figure 8:**
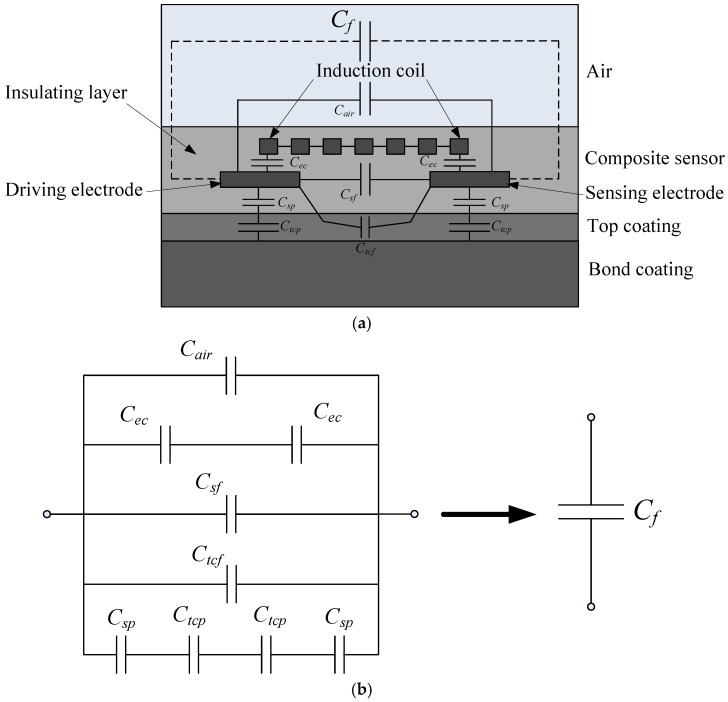
Planar capacitor in the composite sensor for TBC testing: (**a**) schematic sketch in a side view; (**b**) equivalent circuit diagram.

The planar capacitor in the composite sensor for TBCs testing can be described with an equivalent circuit diagram as shown in Figure 8, where *C_air_* is the capacitive contribution of the air, *C_sf_* is the fringe capacitive contribution of the insulating layer in the composite sensor, *C_tcf_* is the fringe capacitive contribution of the top coating, *C_ec_* is the overlap capacitance between the driving electrode and the induction coil, *C_sp_* is the overlap capacitive contribution of the insulating layer between the driving electrode and the metallic bond coating, and *C_tcp_* is the overlap capacitive contribution of the top coating between the driving electrode and the metallic bond coating. The total capacitance of the planar capacitor can be expressed by:

(3) $$C_{f}=C_{air}+\frac{C_{ec}}{2}+C_{sf}+C_{tcf}+\frac{C_{sp}C_{tcp}}{2(C_{sp}+C_{tcp})}.$$

In this capacitor model, the capacitor *C_tcp_* is considerably larger than the capacitor *C_tcf_*. When the thickness of the top coating increases, *C_tcp_* will decrease and *C_tcf_* will increase, while *C_air_*, *C_ec_*, *C_sf_* and *C_sp_* remains constant, and, moreover, the decrease of *C_tcp_* is about two orders larger than the increase of *C_tcf_* [28,29,30,31,32]; thus, the total capacitance *C_f_* will fall with the thickness of top coating. As shown in Figure 8, the capacitance variation against the thickness of the top coating is nonlinear. This is, however, within expectation considering the non uniform field distribution of the planar capacitor since most of the field energy is concentrated around the sensor electrodes.

**Figure 9:**
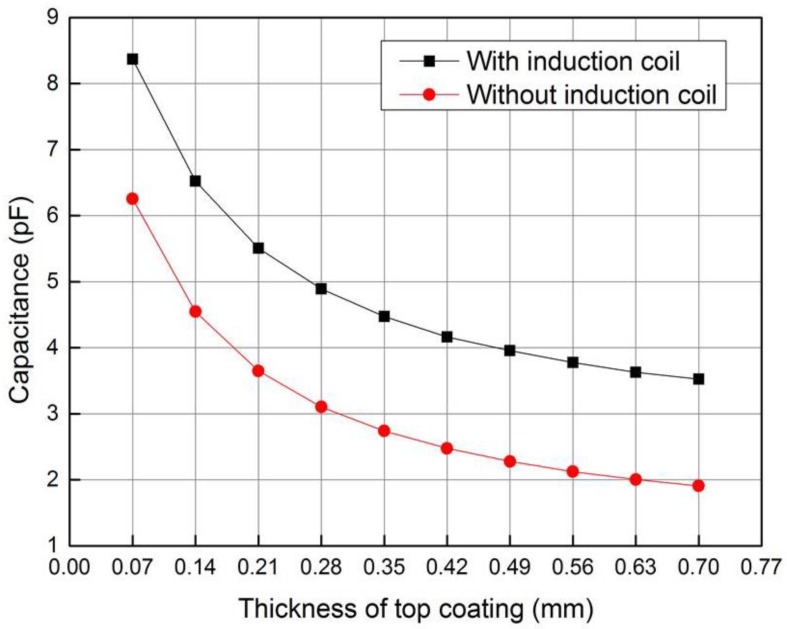
Variation of capacitance with ceramic layer thickness.

It can be seen from Figure 9 that the planar capacitor with the induction coil has higher equivalent capacitance and testing sensitivity to the thickness of ceramic layer than that of the instance without induction coil, which can be explained as following. The voltage applied on the driving electrode will produce charges on the induction coil and the metal bond coating, which will accordingly induce charges on the sensing electrode. The change of charge on the sensing electrode and the driving electrode due to the variety of the ceramic layer thickness is larger than the instance without induction coil. Since the potential difference between the driving electrode and the sensing electrode is constant, the capacitance change and thickness testing sensitivity of planar capacitor is higher when the induction coil exists.

**Figure 10:**
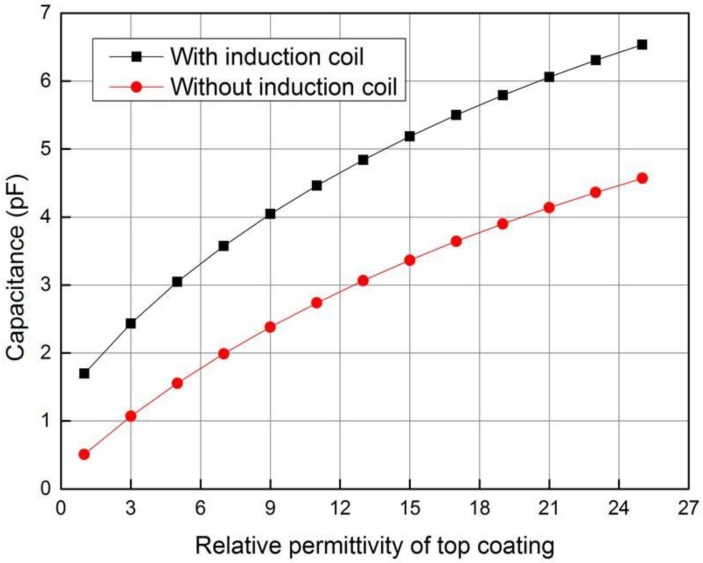
Variation of capacitance with relative permittivity of ceramic layer.

It can be seen from Figure 10 that the capacitance of the planar capacitor will increase nonlinearly with the relative permittivity of the ceramic layer, and the planar capacitor has higher testing sensitivity to the permittivity of the ceramic layer when the induction coil exists. When the induction coil exists and the potential difference between the driving electrode and the sensing electrode is constant, the charge change on the sensing electrode caused by the change of the permittivity of the ceramic layer is larger than the instance without induction coil. Thus, the permittivity testing sensitivity of capacitor is higher.

In a word, the existence of induction coil is beneficial to the sensitivity in capacitive testing mode, and the influence of sensing electrode on the sensitivity of the electromagnetic testing is weaker. Therefore, it is feasible to integrate the electromagnetic unit and the planar capacitor in a sensor and to operate it in time-sharing mode.

## 4. Experimental Validation

**Figure 11:**
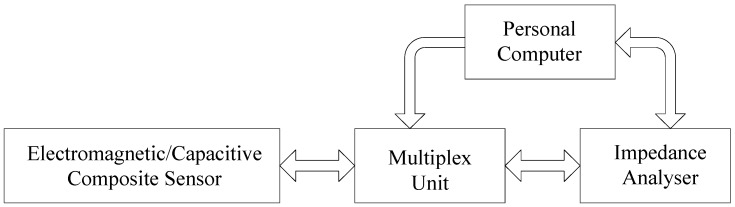
Schematic diagram of the sensor testing system.

In order to verify the effect of the composite sensor, benchmark experiments were carried out. Figure 11 shows the measurement system, which consists of the composite sensor, impedance analyser, multiplex unit, personal computer and a LabVIEW program. A Wayne Kerr 6510B precise impedance analyser (Wayne Kerr Electronics, Chichester, UK) was utilized for capacitance and transimpedance measurement. The operating frequency of the impedance analyser was set to be 100 kHz. This particular frequency ensured that the measurement error of the impedance analyser was less than 0.05% for a 1 pF capacitance, while at the same time giving a good approximation for the electrostatic case in the model for capacitive testing mode. The multiplex unit consists of reed relay switch arrays to extend the number of measurement channels, and to enable different terminal configurations for various measurement protocols. The multiplex unit is connected to *H_CUR_*, *L_CUR_*, *H_POT_* and *L_POT_* terminals of WK 6510B and can switch between the 4-terminal-pair (4TP) and the mutual inductance configurations [33].

**Figure 12:**
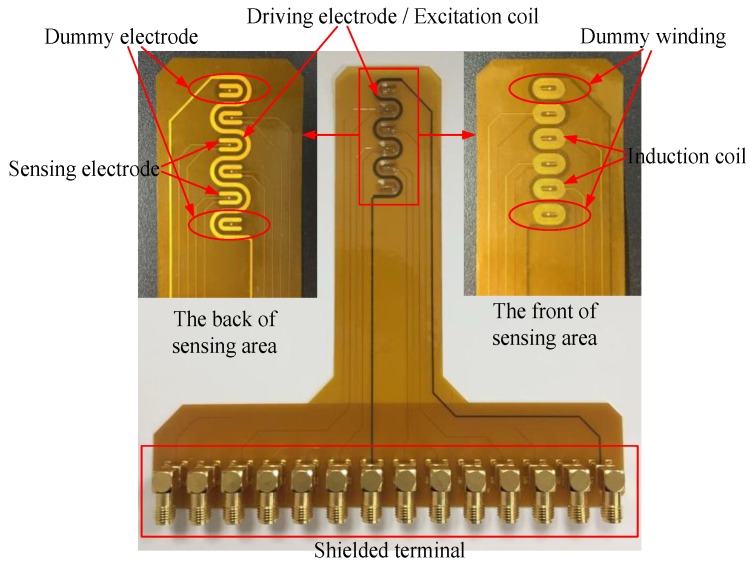
A composite sensor fabricated with flexible printed circuit board (FPCB).

According to the numerical modeling results, we optimized and fabricated the composite sensor for all measurements. As shown in Figure 12, a flexible composite sensor is fabricated with flexible printed circuit board (FPCB) technology. The meandering driving electrode/excitation coil extends a half wavelength at each end of the array, and a pair of dummy sensing elements are formed within those final meander half wavelengths to maintain the periodicity of the field as viewed by the end sensing elements. The dummy elements are not closed and not connected to form a loop so that the net current flowing through the windings is minimized. The purpose of dummy elements is to extend the periodicity of the field beyond the last connected sensing element to reduce the unmodeled “edge” effects at the end of the sensor. The shape of the composite sensor array is T-shaped and the size of sensor is 133 mm × 142 mm. The sensitive area is about 38 mm × 10 mm, including four pairs of detection units and two dummy element structures.

### 4.1. Thickness Testing of Top Coating

A three-layer structure is employed in the experiments as test specimens. The layered structure consists of a plastic slice (as top coating), an Aluminum slice (as bond coating) and a Brass plate (as substrate). The parameters of the test specimens are listed in Table 2.

**Figure 13:**
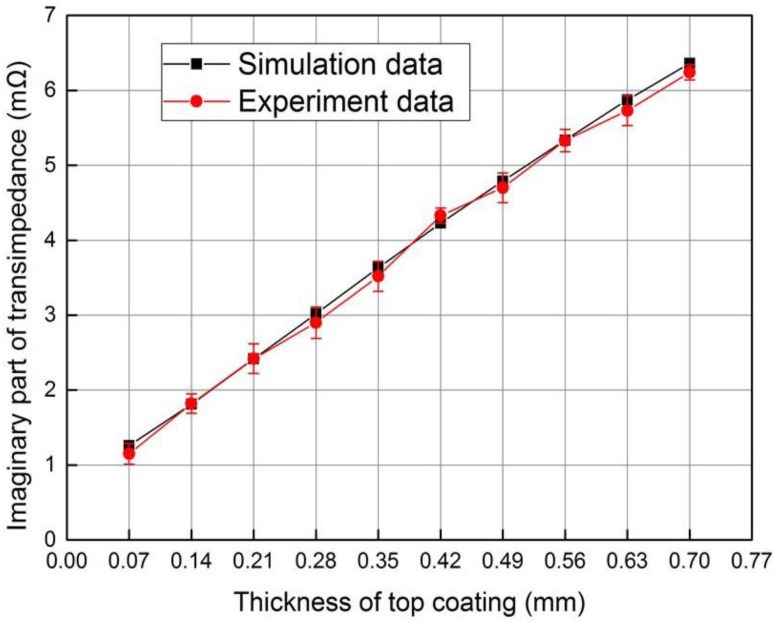
Variety of transimpedance imaginary part with top coating thickness.

The thickness of plastic slice was changed by stacking one to ten thin plastic slices with the same thickness of 0.07 mm. When the composite sensor operates in the electromagnetic testing mode, 20 mA current is applied to the excitation coil with frequency of 100 kHz and the transimpedance of sensor was obtained with the impedance analyser. Figure 13 shows the variety of transimpedance imaginary part with top coating thickness, which shows good agreement with the simulation data.

**Figure 14:**
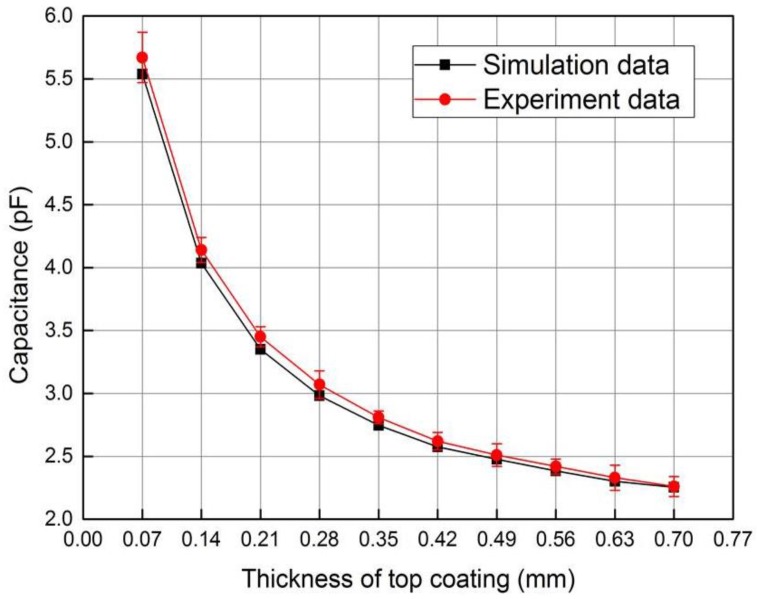
Variety of capacitance with the thickness of top coating.

When the composite sensor operates in the capacitive testing mode, the capacitance of the planar capacitor is measured with the impedance analyzer. Figure 14 shows the variety of capacitance with the thickness of top coating, which shows rather good agreement with the simulation data. Because the simulation model does not consider the influence of parasitic and stray capacitors in the testing circuit, the measured capacitance is slightly larger than the simulation data.

It can be seen from Figure 13 and Figure 14 that the top coating thickness can be tested by both transimpedance and capacitance with impedance analyzer. It is obvious that the composite sensor has higher thickness testing sensitivity in capacitive mode when the thickness is less than 0.21 mm, and the sensitivity is higher and more constant in electromagnetic mode when the thickness is larger than 0.21 mm. In addition, information fusion under two modes can be implemented to improve the reliability of the non-destructive testing (NDT) system.

### 4.2. Conductivity Testing of Bond Coating

The conductivity variety of bond coating will change the voltage of the induction coil, but has no influence on the planar capacitor. Thus, in order to investigate how the sensor signal depends on conductivity of the bond layer, the imaginary part of transimpedance is tabled as a function of the conductivity for a closely spaced set of thicknesses. When the thickness of the top layer and substrate are 0.35 mm and 2 mm, respectively, and two kinds of 0.1 mm thick metal sheet with different conductivities are selected as bond coating, the calculated and measured imaginary part of transimpedance is listed in Table 3, which shows good agreement between the measured value and the calculated value with simulation model for two different values of bond layer conductivity.

In the real coatings, the conductivity change between the bond coating and the substrate is often less than 10%. Therefore, it is difficult to detect and evaluate the changes in the thickness and the conductivity of the bond coating when its conductivity is so close to the substrate.

### 4.3. Permittivity Testing of Top Coating

The capacitance will increase with dielectric permittivity of top coating when its thickness remains constant. In order to investigate how the permittivity affects the sensor signal, the capacitance is tabled as a function of the dielectric permittivity for a closely spaced set of thicknesses. When the thickness of top layer and substrate are 0.35 mm and 2 mm, respectively, the calculated and measured capacitance are listed in Table 4, which shows good agreement between the measured data and the calculated value with simulation model for two different values of top layer permittivity.

## 5. Conclusions

In this paper, an electromagnetic/capacitive composite sensor for comprehensive testing of TBCs is proposed. The ceramic layer can be tested with the planar capacitor, and the metal substrate material can be tested with the electromagnetic coils. The sensor is optimized by simulation analysis, and the interaction between the planar capacitor and the electromagnetic coils is studied. It can be found that the existence of induction coil can improve the sensitivity of planar capacitor to some extent, and the existence of a sensing electrode has a slight negative influence on the sensitivity of the electromagnetic coils. Three kinds of experiments were carried out and the experimental results are in good agreement with the simulation results, which shows the feasibility of integrating the two testing methods. Future work will deal with an attempt to use the experiment data, which measured with the composite sensor in two operating modes, in order to achieve parametric inversion of TBCs.

## Figures and Tables

**Table 1:** Parameters of the thermal barrier coating (TBC) under simulation.

	Thickness (mm)	Conductivity (MS/m)	Relative Permeability	Relative Permittivity
Top coating	0.3	0	1	12.5
Bond coating	0.1	0.2	1	1
Substrate	2	1.6	1	1

**Table 2:** Parameters of the test specimens.

	Thickness (mm)	Conductivity (MS/m)	Relative Permeability	Relative Permittivity
Plastic slice (top coating)	0.07–0.7	0	1	4
Aluminum slice (bond coating)	0.2	38.1	1	1
Brass plate (substrate)	2	58.1	1	1

**Table 3:** Comparison of transimpedance imaginary part between experimental and simulation results.

Conductivity of Bond Layer (MS/m)	Calculated Value (mΩ)	Measured Value (mΩ)	Relative Error (%)
11.4	3.62	3.74	3.31
37.74	3.21	3.31	3.12

**Table 4:** Comparison of capacitance between experimental and simulation results.

Permittivity of Top Layer	Calculated Value (pF)	Measured Value (pF)	Relative Error (%)
2	2.08	2.17	4.32
4.5	2.91	3.02	3.78
